# Is nervus femoralis radiofrequency ablation an effective treatment for recalcitrant greater trochanteric pain syndrome? Results of a cross-sectional study

**DOI:** 10.1093/pm/pnaf153

**Published:** 2025-11-07

**Authors:** Kristen Saad, Chase Young, Amanda N Cooper, Blake Dickenson, Richard Kendall, Allison Glinka Przybysz, Taylor Burnham, Zachary L McCormick, Aaron Conger

**Affiliations:** Department of Physical Medicine and Rehabilitation, University of Utah, Salt Lake City, UT 84132, United States; Department of Anesthesiology, University of Utah, Salt Lake City, UT 84112, United States; Department of Physical Medicine and Rehabilitation, University of Utah, Salt Lake City, UT 84132, United States; Department of Physical Medicine and Rehabilitation, University of Utah, Salt Lake City, UT 84132, United States; Department of Physical Medicine and Rehabilitation, University of Utah, Salt Lake City, UT 84132, United States; Department of Physical Medicine and Rehabilitation, University of Utah, Salt Lake City, UT 84132, United States; Department of Physical Medicine and Rehabilitation, University of Utah, Salt Lake City, UT 84132, United States; Department of Physical Medicine and Rehabilitation, University of Utah, Salt Lake City, UT 84132, United States; Vivo Cura Health, Calgary, AB T2E 2P5, Canada; Department of Clinical Neurosciences, Section of Physical Medicine and Rehabilitation, University of Calgary, Calgary, AB T2N 2T9, Canada; Department of Physical Medicine and Rehabilitation, University of Utah, Salt Lake City, UT 84132, United States; Department of Physical Medicine and Rehabilitation, University of Utah, Salt Lake City, UT 84132, United States

**Keywords:** nervus femoralis, radiofrequency ablation, greater trochanteric pain syndrome, lateral hip pain

## Abstract

**Objective:**

To describe long-term treatment outcomes of nervus femoralis radiofrequency ablation (NF-RFA) for recalcitrant greater trochanteric pain syndrome (GTPS).

**Design:**

A cross-sectional study.

**Methods:**

Chart review of consecutive patients who underwent NF-RFA from 2022 to 2023 was performed. A standardized telephone survey was utilized to capture current Numeric Pain Rating Scale (NPRS) and Patient Global Impression of Change (PGIC) scores. The primary outcome was ≥50% NPRS score reduction at follow-up. A secondary analysis was completed on free text responses asking patients to describe post-procedural changes in pain and function in their own words.

**Results:**

Outcomes were collected from 25 patients (aged 71.7 ± 9.3 years; 80.0% female; body mass index 29.3 ± 6.8 kg/m^2^) for 27 NF-RFA procedures at a minimum follow-up time of 6 months post-procedure. Average follow-up time was 13.1 ± 4.9 months; ≥50% NPRS reduction from baseline was reported by 55.6% (*n *= 15/27; 95% CI, 37.3-72.4) of patients. In addition,  ≥ 2-point NPRS score reduction from baseline was reported by 70.4% (*n *= 19/27; 95% CI, 51.5-84.2) of patients, and 51.9% (*n *= 14/27; 95% CI, 34.0-69.3) reported a PGIC score consistent with “much improved” or “very much improved.”

**Conclusion:**

In this cohort, over 55% of patients who received NF-RFA as treatment for refractory GTPS reported at least 50% improvement in hip pain at an average follow-up of approximately 13 months. The majority of free text responses from patients indicated that they would recommend NF-RFA, while approximately 25% reported ongoing pain and disability from low back pain or a return of index hip pain symptoms post-procedure.

## Teaser

This cross-sectional study evaluated long-term (6+ month) outcomes of nervus femoralis radiofrequency ablation (NF-RFA) for patients with refractory greater trochanteric pain syndrome (GTPS). After an average of 13.1 months, over half of patients reported at least a 50% reduction in pain. While the majority of patients indicated they would recommend the procedure, about 25% continued to experience pain or recurrence of symptoms.

## Introduction

Greater trochanteric pain syndrome (GTPS) is a prevalent condition characterized by lateral hip pain that has been estimated to have a lifetime prevalence of 10% to 25%.^1^ GTPS significantly impairs quality of life and has been associated with lower health status, lower pressure pain threshold, reduced hip abductor/extensor strength, increased body mass index (BMI),^2^ and increased depression and anxiety.^3^ Advances in understanding its pathophysiology have shifted the diagnostic focus from trochanteric bursitis to include gluteal tendinopathies, among other causes.^4^ This evolution has led to diverse nonoperative management strategies, including injections, shockwave treatment, and physical therapy, though many cases of GTPS remain recalcitrant to these treatments.^4^ Given the prevalence and impact of this condition, as well as the paucity of treatment options in refractory cases, there is a critical need for research to develop more effective interventions for treatment resistant cases of GTPS.

The sensory innervation of the greater trochanter and its surrounding structures may represent a target for the treatment of GTPS. A study by Genth et al. revealed a small sensory nerve supply to the periosteum and bursae of the greater trochanter, named the Nervus Femoralis (NF).^5^ This sensory nerve, a branch of the femoral nerve, accompanies the arteria and vena circumflexa femoris medialis and their trochanteric branches to the greater trochanter, entering the periosteum directly caudal to the tendon of the inferior gemellus muscle. Furthermore, anatomic work has shown significant sensory innervation of the superficial fascial structures around the hip in this same region.^6^ This understanding of the specific neural pathways involved in lateral hip nociception has led investigators to propose targeted denervation strategies to reduce pain and disability associated with GTPS.^7^

Radiofrequency ablation (RFA), a minimally invasive procedure that uses thermal energy to coagulate tissue, is commonly used to accomplish targeted denervation for various spine and musculoskeletal conditions.^8^ Its safety in peripheral joint denervation has been demonstrated to by comparable to intra-articular injection, even in cases where nerves run in close proximity to vasculature, such as the genicular nerves around the knee.^9^

The use of RFA for the alleviation of GTPS has been proposed by interrupting nociceptive signals via neurolysis of the nervus femoralis. Abd-Elsayed et al. published a case series of 8 patients reporting an average pain reduction of 71.4% at 16-169 days after the use of nervus femoralis radiofrequency ablation (NF-RFA) using internally cooled technology.^10^ Vajdi et al. reported the case of a single patient who experienced significant relief following cooled NF-RFA to treat GTPS recalcitrant to conservative and surgical interventions including trochanteric bursectomy and iliotibial band lengthening.^11^ Others have targeted this nerve with non-ablative radiofrequency treatment.^12^ Vieira et al. treated 9 patients with bipolar pulsed RF of the nervus femoralis and reported an average score reduction of 76.5% on the Brief Pain Inventory—Short Form (BPI-SF) at 6 months, with all but one participant achieving greater than 50% pain relief.^12^

Given the published proof of concept results,^10^^,^^12^ we aimed to further characterize the effectiveness of NF-RFA in a larger patient sample with recalcitrant lateral hip pain attributable to GTPS.

## Methods

### Data collection

This study was approved by the University of Utah Institutional Review Board (IRB_00138414) and was conducted at a single, tertiary academic spine center. Medical records of patients who underwent nervus femoralis RFA between May 2022 and December 2023 were reviewed. Inclusion criteria for patients were (1) age ≥ 18 years and (2) NF-RFA at least 6 months prior to follow-up contact. At our institution those who were considered for NF-block/RFA were those with persistent lateral hip pain consistent with GTPS who had failed to improve with conservative measures typically including physical therapy, oral analgesics, and targeted corticosteroid injection. Data collected from electronic medical records included age, gender, body mass index, and radiofrequency probe type.

Long-term outcomes (≥6 months post-RFA) were collected via a standardized telephone survey, which included Numeric Pain Rating Scale (NPRS) pain scores and Patient Global Impression of Change (PGIC) self-reported improvement metrics. Additional qualitative measures were assessed, including patient willingness to recommend the procedure to a friend or family member, and free text descriptions of changes in pain and function as a result of the procedure. The primary outcome was the proportion of patients who reported ≥50% NPRS reduction of index pain. Secondary outcomes examined treatment success as measured by (1) the proportion of patients who reported ≥2-point NRS score reduction from baseline, representing the minimally clinically important change (MCIC),^13^ and (2) the proportion of patients who reported a PGIC score of 6 or 7 consistent with a rating of at least “much improved.”

To assess qualitative response to treatment, patients were asked if they would recommend NF-RFA to a friend or family member (yes/no) as a part of the standardized telephone survey. Additionally, patients were given the opportunity to describe post-procedure changes in pain and/or function in their own words, and their responses were recorded in the survey database as free text.

### Procedures

Procedures were performed by Physical Medicine and Rehabilitation physicians with sub-specialty fellowship training in either Pain Medicine or Interventional Spine and Musculoskeletal Medicine.

### Nervus femoralis blocks

**Figure 1.**
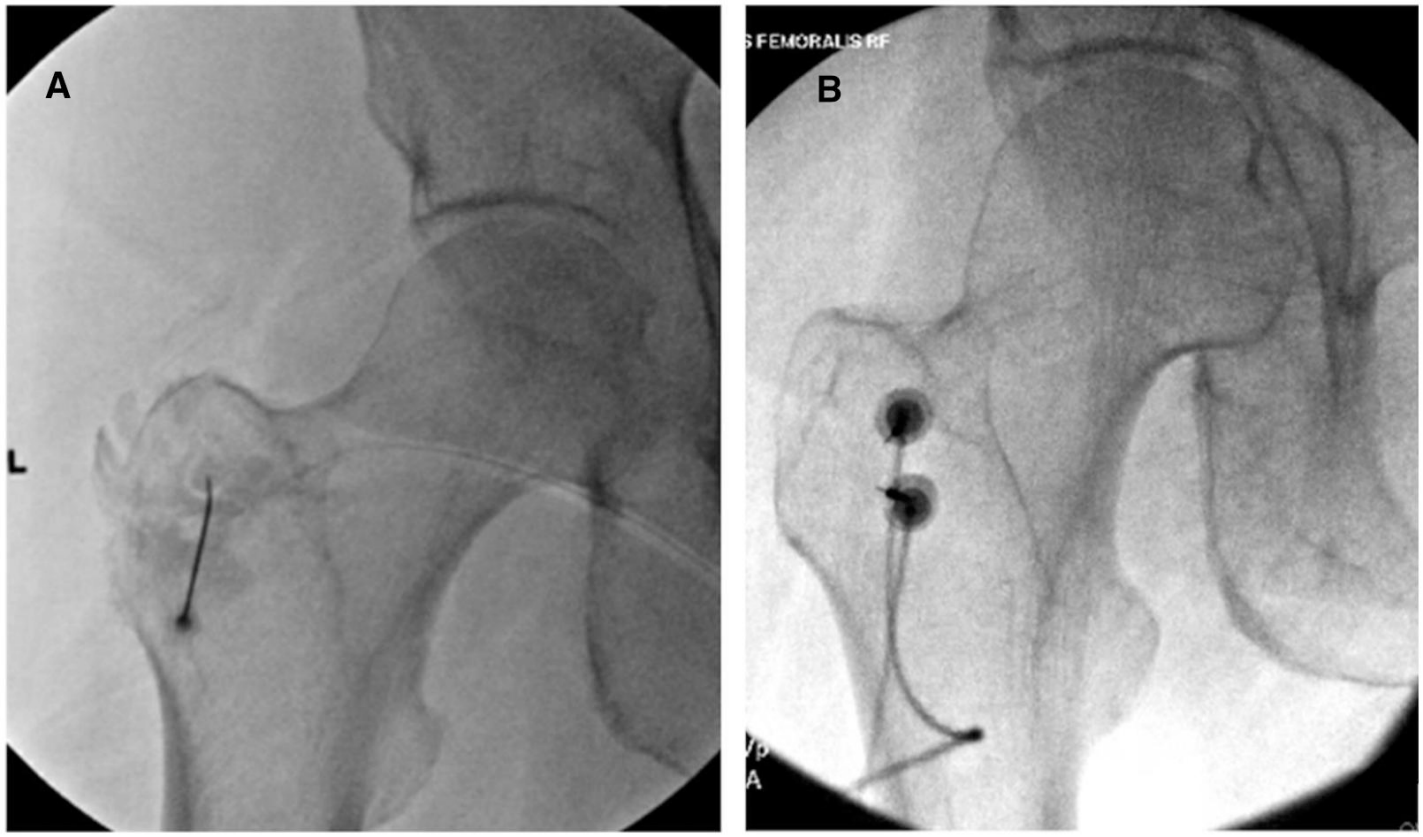
Fluoroscopic views of (A) needle positioning for pre-procedural diagnostic block of the nervus femoralis and (B) positioning of electrodes to complete nervus femoralis radiofrequency ablation.

Participants were brought into the procedure room and positioned prone on the procedure table. The skin was prepped and draped in the usual sterile fashion with betadine or chlorhexidine. The posterior aspect of the greater trochanter was identified under fluoroscopy in neutral hip alignment. The skin and subcutaneous tissues were then anesthetized with 1-2 mL of 1% lidocaine. Under direct fluoroscopic guidance, a 3.5-5 inch, 22-gauge or 25-gauge spinal needle was then directed to the known location of the NF based upon cadaveric and published technical protocols (Figure 1).^5^^,^^7^ Isovue (or Prohance in the case of iodinated contrast allergy) was subsequently injected to confirm spread intended to cover the known location of the traversing NF, after which 1 mL of 4% lidocaine or 0.5% bupivacaine was injected.

Patients were asked to report the change in their lateral hip pain after the procedure. The diagnostic block was considered successful if it resulted in 50% or greater reduction in their index pain.

### Nervus femoralis radiofrequency ablation

For NF-RFA, participants were positioned, prepared, and the soft tissue anesthetized in the same fashion as described for the block procedure (as above). Under direct fluoroscopic guidance, radiofrequency introducer needles were directed toward the known location of the NF. Needle positioning was confirmed in the hip-neutral AP view by contacting the bony backstop of the greater trochanter, ensuring appropriate depth and medial-lateral orientation (Figure 1). For multi-tined canulae, the tines were then deployed. Following injection of lidocaine, lesions were performed at 85 °C for 180 s at each site for bipolar RFA techniques, 80 °C for 90 s for conventional monopolar RFA techniques, and 60° (with internal lesion temperature of 80°) for 165 s for internally cooled monopolar RFA techniques. Cannula length varied based on patient habitus, ranging from 50 to 150 mm. The following electrode types were used: Conventional 18G cannulae with 10 mm active tips, internally cooled 17G probes with 4 mm tips, dual-tined 18G electrodes with 10 mm tips, and 3-tined 18G electrodes with 5 mm tips. Following ablation, all electrodes were removed, and the skin entry sites were covered with bandages.

### Statistical analysis

Patient demographics and clinical characteristics were summarized using descriptive statistics, with calculation of means/standard deviations (SDs) for continuous variables and frequencies/percentages for categorical variables. A 95% confidence interval (CI) was also calculated for the statistics of select variables, including all primary and secondary categorical outcome variables. Logistic regression analysis was performed to explore the associations between treatment success as measured by NRS and PGIC and select predictor variables, with calculation of odds ratios (ORs) and their 95% confidence intervals.

### Qualitative analysis

Word frequency analysis was completed on open-ended responses describing changes in pain and/or function as a result of NF-RFA using automated text mining tools. Data were imported into RStudio^14^ and analyzed using R (version 4.3.0).^15^ Free text responses were then cleansed to remove special characters and numbers, and normalized to remove contractions and standardize capitalization. Stop words such as “the” and “and” were removed via filtering using a standardized lexicon. The TidyText^16^ package was subsequently used to segment the responses into single-word tokens, which were then aggregated by frequency.

Open-ended responses from patients were further manually reviewed in Microsoft Excel to identify several overall themes. The presence of themes was independently graded by 3 reviewers (K.S., C.Y., and A.N.C.), with a positive thematic identification requiring the assent of at least 2 reviewers.

## Results

Forty patients met study criteria. Two patients declined to participate. One patient passed away of unrelated causes prior to contact, and 12 patients could not be contacted. Twenty-five patients, who underwent a total of 27 NF-RFA procedures, consented to participate and were included in the analysis. Participant demographics, clinical characteristics, and procedural variables are shown in Table 1. The majority of participants were female (80.0%) with a mean age of 71.7 ± 9.3 years and mean BMI of 29.3 ± 6.8 kg/m^2^. Primary and secondary categorical outcomes are summarized in Table 2. At a mean follow-up time of 13.1 ± 4.9 months, ≥50% NRS reduction (the primary study outcome) was reported by 55.6% of participants (*n *= 15/27; 95% CI, 37.3-72.4), with 70.4% of participants (*n *= 19/27; 95% CI, 51.5-84.2) reporting a ≥ 2-point NRS score decrease. The proportion of participants with a PGIC score ≥6 (corresponding to a self-assessed rating of overall change as “much improved” or “very much improved”) was 51.9% (*n *= 14/27; 95% CI, 34.0-69.3). Results of the logistic regression analysis are presented in Table 3. The 2 chosen predictor variables, RFA probe type and BMI, were not significantly associated with either ≥50% or ≥2-point NRS reduction at follow-up. However, the regression model for ≥6 on PGIC identified BMI as a significant predictor variable, with the odds of a successful outcome found to decrease by 25% for each additional kg/m^2^.

**Table 1.** Participant demographics, clinical characteristics, and procedural variables.

Variable	No. (%)
Follow-up time period (*n *= 27)	
6-12 months	13 (48.1)
12-24 months	13 (48.1)
≥24 months	1 (3.7)
Gender (*n *= 25)	
Female	20 (80.0)
Male	5 (20.0)
Current smoking (*n *= 25)	
Yes	3 (12.0)
No	22 (88.0)
History of total or partial joint replacement in index hip (*n *= 25)	
Yes	3 (12.0)
No	22 (88.0)
Anxiolytic/antidepressant medication use (*n *= 25)	
Yes	9 (36.0)
No	16 (64.0)
Diagnostic block pain relief (*n *= 27)	
50%-79%	3 (11.1)
80%-99%	5 (18.5)
100%	19 (70.4)
NF-RFA probe type (*n *= 27)	
Conventional	1 (3.7)
Internally cooled	3 (11.1)
Dual-tined	18 (66.7)
Three-tined	5 (18.5)
NF-RFA laterality (*n *= 25)	
Left	11 (44.0)
Right	12 (48.0)
Bilateral	2 (8.0)
History of prior NF-RFA in index hip (*n *= 25)	
Yes	0 (0.0)
No	25 (100.0)
Age in years (*n *= 25), mean (SD)	71.7 (9.3)
BMI in kg/m^2^ (*n *= 25), mean (SD)	29.3 (6.8)
Duration of pain in years (*n *= 9), mean (SD)	13.6 (18.6)
Follow-up time in months (*n *= 27), mean (SD)	13.1 (4.9)

Abbreviations: NF-RFA, nervus femoralis radiofrequency ablation; SD, standard deviation.

**Table 2.** Primary and secondary outcomes.

	No. (%)		
Outcome	Yes	No	95% CI (yes)
≥50% NRS reduction	15 (55.6)	12 (44.4)	37.3, 72.4
≥2-point NRS reduction	19 (70.4)	8 (29.6)	51.5, 84.2
≥6 on PGIC	14 (51.9)	13 (48.1)	34.0, 69.3

Abbreviations: CI, confidence interval; NRS, numeric rating scale; PGIC, Patient Global Impression of Change.

**Table 3.** Logistic regression models for ≥50% NRS reduction, ≥2-point NRS reduction, and ≥6 on PGIC.

Outcome	Predictor	OR	95% CI	*P*
≥50% NRS reduction^a^	**Probe type** (vs internally cooled)			
	Dual-tined	3.23	0.06, 9.64	0.82
	Three-tined	8.08	0.56, 115.99	0.12
	**BMI**	1.12	0.98, 1.28	0.09
≥2-point NRS reduction^b^	**Probe type** (vs internally cooled)			
	Dual-tined	0.75	0.06, 9.64	0.82
	Three-tined	2.90	0.10, 87.48	0.54
	**BMI**	1.07	0.95, 1.22	0.26
≥6 on PGIC^c^	**Probe type** (vs internally cooled)			
	Dual-tined	0.15	0.02, 1.33	0.09
	Three-tined	0.13	0.00, 4.33	0.26
	**BMI**	0.75	0.60, 0.95	0.02*

Abbreviations: CI, confidence interval; NRS, numeric rating scale; OR, odds ratio; PGIC, Patient Global Impression of Change.

* denotes statistically significant.

a *N *= 24; *χ*^2^ (3) = 4.40; *P *= 0.22; Pseudo *R*^2^ = 0.09.

b *N *= 24; *χ*^2^ (3) = 1.68; *P *= 0.64; Pseudo *R*^2^ = 0.05.

c *N *= 24; *χ*^2^ (3) = 6.52; *P *= 0.09; Pseudo *R*^2^ = 0.31.

### Qualitative analysis

**Figure 2.**
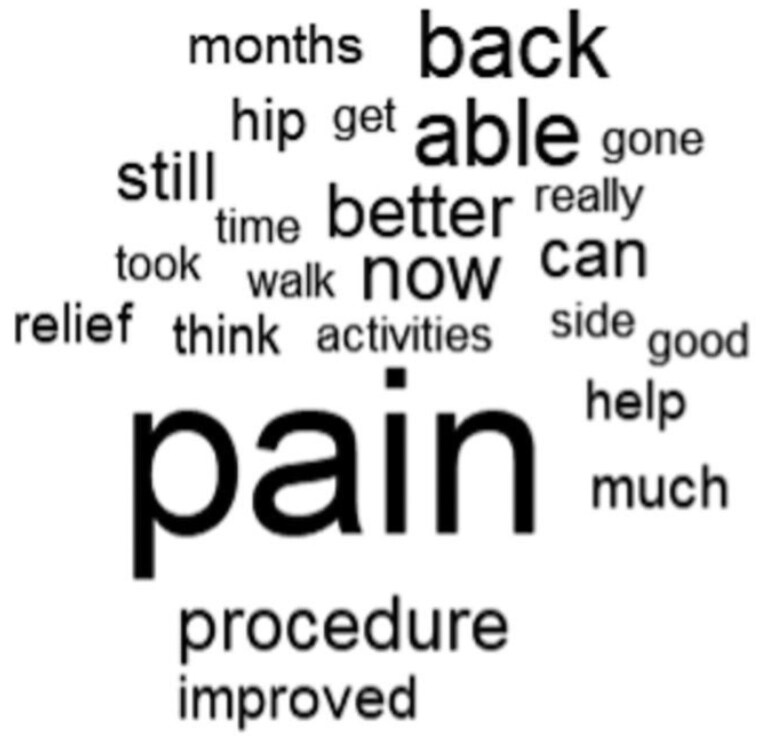
Word cloud generated from patient descriptions of changes in pain and/or function following nervus femoralis radiofrequency ablation (NF-RFA). Words are scaled by frequency of occurrence (minimum 3 occurrences for inclusion). The size of each word corresponds to its relative frequency in patient reports.

The word frequency analysis revealed that “back,” “month,” and “walk” occurred commonly, in addition to expected words such as “pain,” “procedure,” and “hip.” A word cloud was generated to visualize words used at least 3 times, and scaled linearly by number of occurrences (Figure 2).

When manually reviewing open-ended patient descriptions of change in pain and/or function as a result of NF-RFA, several patterns were observed (Table 4); 28% (7/25) of patients described a limited duration of relief following previous improvement, typically endorsing weeks-to-months of relief with subsequent return of lateral hip pain. In addition, 20% of patients (5/25) expressed concerns related to concomitant, complicating back pain; 9/25 (36%) patients were able to identify functional improvement in specific activities, such as gardening, hiking, and walking, following the procedure; 76% of patients (19/25) stated they would recommend NF-RFA to a friend or family member.

**Table 4.** Themes identified from patient-provided free text responses describing changes to pain and function following NF-RFA.

Theme, *n* (%)	Responses (*N* = 25)
Duration of relief	7 (28)
Concomitant back pain	5 (20)
Functional improvement in at least 1 named, specific activity	9 (36)

Abbreviation: NF-RFA, nervus femoralis radiofrequency ablation.

## Discussion

In this cross-sectional study, we observed clinically significant reductions in greater trochanteric pain in over half of participants treated with NF-RFA. At a mean follow-up of 13.1 months, 55.6% of participants reported ≥50% NPRS reduction, while 70.4% reported ≥2-point NPRS reduction. Furthermore, 51.9% of participants reported that their overall health status was either “much improved” or “very much improved” since their NF-RFA procedure, as denoted by scores ≥6 on the PGIC. We also identified BMI as a significant negative predictor variable as defined by the PGIC: patients with higher BMIs had a decreased likelihood of reporting ≥6 on PGIC at follow-up. When asking patients whether they would recommend this procedure to a friend or family member, an overwhelming majority of patients indicated they felt the risk–benefit balance of the procedure was sufficient to recommend it to a loved one even if they no longer felt relief from NF-RFA.

While the landmark Abd-Elsayed NF-RFA case series (2022)^10^ reported that 6/8 participants (75%) experienced ≥50% pain score reduction and 8/8 (100%) ≥2 pain score reduction, follow-up times for that study were 16-169 days, which is considerably shorter than our study which only selected participants at least 6 months (∼180 days) post-procedure (average follow-up time of 13.1 months or ∼398 days). Our population was also significantly older (71.7 ± 9.3 years) compared to the original case series (48 ± 9.7 years). Average patient BMIs were not significantly different.^10^

Notably, the Abd-Elsayed case series stated that all 8 patients had a history of chronic axial low back pain in addition to GTPS,^10^ and 20% of the patients in our study felt concomitant back pain was limiting enough to them to independently report it in open-ended responses describing pain and function post NF-RFA. This warrants further consideration of potential co-treatment options that may address underlying biomechanical deficiencies and pain sensitization.

BMI has been shown to be associated with GTPS, likely due to increased stress and biomechanical imbalances across the gluteal tendons.^2^^,^^17^ Thus, it is not necessarily surprising that our analysis revealed a negative association between BMI and NF-RFA procedural response. Larger studies would be elucidating as to the strength of this relationship. Physical therapy and weight management have long been cornerstone conservative treatments of GTPS,^17^ and this finding emphasizes their continued importance as part of a multimodal treatment plan.

Finally, while a significant proportion of participants reported ongoing relief of their lateral hip pain following NF-RFA with an average follow-up time of 13.1 months, 28% reported pain that returned after a period of initial improvement. This is important given that this positive response would not necessarily be captured in pain score or PGIC outcomes, but potentially would have been captured with earlier follow-up time. It is well-understood that nerves have the potential to regenerate after RFA,^10^^,^^18^ which can be associated with a return of pain. Future studies may consider following up at more standardized intervals to better understand duration of relief when denervating the greater trochanter and related structures.

### Limitations

This study notably has several limitations. First, the retrospective, cross-sectional design of the study is less robust compared to a prospective study with planned follow-up times. Additionally, no control group was utilized to compare outcomes. Changes in functional and PROMIS scores could not be computed due to lack of systematic inclusion in pre-procedural documentation. Finally, due to the relative rarity of use of NF-RFA at our institution, the overall sample size was relatively low, which reduces statistical power for reporting outcomes based on patient demographics, clinical characteristics, RFA probe type, and other factors. This was further complicated by low response rate to survey and contact prior to inclusion window of 6 months.

One of the persisting challenges of treating GTPS is that anatomy and pathophysiology remain incompletely understood. While anatomic studies by Genth et al.^5^ have helped develop a better understanding of structural innervation, these remain somewhat limited. Additionally, myofascial overlay, concomitant tendinopathic processes, and central and peripheral sensitization may all contribute to chronic lateral hip pain and may not be completely addressed by radiofrequency ablation of sensory nerves specifically innervating the greater trochanter.

Additional studies would be beneficial in more fully elucidating specific sources of pain in GTPS. In addition to further anatomic and cadaveric studies that could enhance our understanding of regional innervation, future prospective and randomized clinical studies could shed light on other factors influencing clinical response, including back pain, BMI, and baseline level of function.

## Conclusions

In this cross-sectional study, over 55% of participants selected by diagnostic nervus femoralis blocks with ≥50% pain relief reported a ≥ 50% reduction in recalcitrant greater trochanteric pain at an average of 13 months after nervus femoralis RFA; 76% of participants said they would recommend the procedure to their friends and family, which reflects positive views of overall procedural risks and costs versus benefits. This study, the largest investigating ablative treatment of the nervus femoralis for treating recalcitrant lateral hip pain, further supports the utilization of nervus femoralis radiofrequency ablation to treat GTPS.
